# Integration experiences of student and qualified nurses with disabilities who graduated from selected KwaZulu-Natal nursing education institutions: An exploratory case study

**DOI:** 10.4102/curationis.v41i1.1862

**Published:** 2018-08-23

**Authors:** Selvarani Moodley, Gugu Mchunu

**Affiliations:** 1 KwaZulu-Natal College of Nursing, Pietermaritzburg, South Africa; 2 School of Nursing and Public Health, University of KwaZulu-Natal, South Africa

## Abstract

**Background:**

Despite the introduction of the *Disability Policy Guidelines* in South Africa (SA), student nurses who have disabilities (SNWDs) are still habitually and effectively excluded from nurse training programmes, and hence from the nursing profession. Yet SNWD may be able to offer a unique perspective.

**Objectives:**

To explore and describe the integration experiences of both student and qualified nurses with disabilities who graduated from selected KwaZulu-Natal nursing education institutions (NEIs).

**Method:**

A concurrent mixed-method design based on multiple embedded case studies served as the primary data collection instrument in this paper. The researcher initially conducted a survey of all the private NEIs to determine which had experienced training SNWDs; 3 cases and 10 embedded cases were selected, using non-probability purposive sampling. Individual interviews were conducted with students and qualified nurses with who have a disability (*n* = 10) who had graduated from NEIs.

**Results:**

The findings of the study indicated that, largely, private NEIs are paving the way for integrating SNWDs; however, there are still some gaps in meeting the needs of these students. Despite strong legislative policies, an inclusive and enabling teaching and learning environment for SNWDs in nurse training remains largely absent.

**Conclusion:**

The study recommends that NEIs develop policy guidelines for integrating SNWDs in nursing education programmes promoting an inclusive nursing education for SNWDs. The introduction of a disability liaison to assist SNWDs by liaising between key stakeholders and, perhaps, addressing many of the challenges that SNWDs experience in the clinical facilities where nursing personnel are unaware of their disability.

## Background

It is estimated that more than one billion people are living with some form of physical, sensory, intellectual or mental disability (UNAIDS 2014). According to Statistics SA, the national prevalence of disability is 7.7%. It is more prevalent in female than in male gender (8.9% and 6.5%, respectively). In 2015, 90% of nurses registered with the South African Nursing Council (SANC) were female and 10% were male (*n* = 148 782) (SANC 2015), confirming that nursing is a female-dominated profession with increased disability prevalence. Disability prevalence is also proportional to increase in age, which has further implications for nursing (World Health Organization 2011). Despite global policies such as the *Americans with Disability Act* and the United Nations Convention on the Rights of Persons with Disabilities (UNCRPD) prioritising the right of people with disability to be included in the general education system, removal of barriers in the environment and provision of support and reasonable accommodation (United Nations Human Rights 2008), in SA, only one fifth (5.3%) of people living with severe disabilities attend tertiary education institutions to obtain a post-school qualification (Statistics South Africa 2016).

South Africa (SA) is one of 20 countries that ratified the UNCRPD in 2008, which confirmed that all persons with disabilities should enjoy equal human rights and freedoms (National Department of Health 2015), as is echoed by the Constitution of South Africa (Republic of South Africa 1996). This means that disabled people may not be discriminated against and should, therefore, have equal opportunities to undertake nurse training without discrimination (Hargreaves et al. 2014). However, a recent study by Neal-Boylan and Smith (2016) confirms that discrimination against student nurses who have disabilities (SNWDs) in nursing programmes still occurs. Despite regulations promoting inclusion of SNWDs, a survey conducted in three universities in SA indicated that they represented only 0.5% of the student population (Crous 2004), suggesting that they continue to be underrepresented in nurse training (Marks & Ailey 2014). A valid explanation for this underrepresentation could be the lack of disclosure by students with invisible disabilities as a result of the stigma attached to it. Students are sometimes forced to disclose their disability when they experience challenges during training and may thus require some form of reasonable accommodation. While some students are born with disabilities, some become disabled prior to commencement of their training, but others do so during training. As excluding these students from the nurse training programme would result in discriminatory practices, nursing education institutions (NEIs) are faced with the challenge of providing support and reasonable accommodation for SNWDs in the absence of clear policy guidelines.

The White Paper on the rights of persons with disabilities in SA describes reasonable accommodation as ‘necessary and appropriate modification and adjustments, including assistive devices and technology, not imposing a situation, to ensure persons with disabilities enjoy or exercise on an equal basis with others’ (South Africa National Department of Health 2015). However, reasonable accommodation may vary among the different NEIs, especially in the absence of clear policy guidelines. One of the ways to invoke reasonable accommodation includes disclosure by the students themselves while in some such instances, SNWDs are requested to present a medical certificate confirming the disability. Previous study findings have, however, revealed lack of disclosure (Hargreaves et al. 2014) because of stigma attached to disability (De Cesarei 2014), and Howell (2006) adds that students who have disabilities (SWDs) have experienced lack of reasonable accommodation and negative attitudes as disabling.

Despite the global increase in the number of SWDs in higher education, they still face many challenges, such as structural (physical access), cultural and attitudinal difficulties resulting from ignorance (Disability Rights Commission 2007). Highlighted experiences include physical access barriers and difficulty in accessing lecture venues (Hall & Belch 2000). Fitchett (2015) concludes that this could be because of past practices of building universities to meet the needs of non-disabled students without considering SWDs. Physical access to higher education institutions and lack of transport to fieldwork sites are additional challenges (Khuzwayo 2011; Ndlovu & Walton 2016).

Attitudinal challenges are highlighted by Dahl (2010) that educators of baccalaureate nursing students lacked knowledge and experience in educating and training SNWDs in the classroom and during clinical placements. This resulted in oppressive behaviour, such as requesting SNWDs to perform a ‘pre-skill’ that their peers were not required to perform. While SNWDs can manage the academic environment because of the readily available support while attending lectures, the challenges experienced by SNWDs are increased by the clinical expectations of the nurse training programme and they find the clinical environment unprepared as a result of the lack of policies that guide their practice and clinical accompaniment (Ashcroft & Lutfiyya 2013; Mutanga 2017).

The widespread misconception about the abilities of people with disabilities applies equally in the nursing profession (Neal-Boylan 2013). Nurses with disabilities are seldom given the opportunity to prove their abilities, because of the assumption that they need to take care of themselves (Neal-Boylan 2013) – an assumption that stems from the medical model of disability. This model, also referred to as the deficit model, emphasises disability as inherent in an individual (Barnes & Mercer 2010), which is then extended to the capabilities of an individual being seen as contingent upon the impairment. Hence, disability is still perceived as a medical phenomenon, measuring illness, deviation or dependence within the health profession, rather than it being seen as a matter of equality. This perception disables people in the nursing profession (Scullion 2010). In the present study, the approach is guided, instead, by critical disability theory and the social model of disability, acknowledging it as a social construct (Shakespeare & Watson 2002). In this model, disability is seen as socially constructed in response to barriers in the environment, such that it is the physical environment that impedes a person and not the internal disability itself (Oliver & Barnes 2010).

There is a paucity of current empirical research regarding SNWDs in nursing education. Hence, higher education institutions struggle to understand their experiences and specific examples are needed of graduating SNWDs in the nursing profession (Mertens 2012) to enable nurse educators and NEIs to provide adequate support and reasonable accommodation to meet their needs.

## Problem statement

Since the transformation of the education sector in 1994, increasing numbers of SWDs access higher education (Bell, Carl & Swart 2016). There remains a limited body of published studies on SWDs in these institutions in SA (Mutanga 2017) and various reasons could be offered for this lack, such as little reliable data on disabled people, coupled with the lack of consensus on the meaning of disability (Foundation of Tertiarty institutions of the Northern Metropolis 2011). The stigma attached to disability is an added challenge for SWDs, often resulting in their lack of disability disclosure. While some recommendations have been made to accommodate and include SNWDs in nurse training programmes globally (Holloway 2001), little research has been conducted in SA exploring the actual experiences of students graduating in professions such as nursing (Ndlovu & Walton 2016). Hence, the author of this research saw the necessity to give a first-hand account of SNWDs in nursing programmes to inform policy guideline development for integrating SNWDs into nurse training.

## Research purpose

The purpose of this research is to explore and describe the experiences of student and qualified nurses with disabilities who have graduated from selected NEIs in KwaZulu-Natal.

### Definition of key concepts

*Disability*: This is an umbrella term for impairments, activity limitations and participation restrictions, denoting the negative aspects of the interaction between an individual (with a health condition) and contextual factors (environmental and personal) affecting that individual (United Nations Human Rights 2008). In this study, a student who has a disability is described as having a physical or mental impairment that limits one or more life activities, resulting in participation restrictions. It includes both students with congenital disabilities and those who become disabled prior to commencement or during the nurse training programme.

### Research method and design

A qualitative design using the case study approach was utilised to attain the objectives of the study. Qualitative designs are employed to do an in-depth investigation of a phenomenon of interest (Botma et al. 2010:182), while case studies are used to explore a programme, event or activity of one or more individuals in depth (Yin 2014). The central question was, ‘How have SNWDs and qualified nurses who graduated from KwaZulu-Natal NEIs experienced being in a nurse training programme?’. The findings presented here are part of a larger doctoral study aimed at developing policy guidelines for integrating SNWDs into nurse training programmes.

### Population and sampling

The population comprised all SNWDs currently in nurse training and qualified nurses who completed their training within 2 years and who had graduated from KwaZulu-Natal NEIs. Purposive sampling was used to select student and graduated nurses with disabilities who met the following inclusion criteria (Polit & Beck 2014:179):

- had a disability, regardless of the type
- were registered as a learner at a private NEI in KwaZulu-Natal
- nurses with disabilities who registered as learners at a NEI and who had graduated within 2 years after completion of training.

### Case study protocol

A case study protocol increases the reliability of case study research (Yin 2014:84). This study was guided by a protocol which incorporated the research instrument and general rules for its implementation:

### Case description

Four NEIs met the inclusion criteria for participation and were the cases in this study. The SNWDs, educators and activities of these four cases were the embedded cases.

Case 1 included one public university in KwaZulu-Natal offering the R425 course (Diploma in Nursing [General, Community, Psychiatry] and Midwifery).

Case 2 included three different private NEIs from the same hospital-based groups. Two students were registered in the R2175 (course leading to enrolment as a nurse) and one in the R683 (Bridging course for enrolled nurse leading to registration as a General Nurse).

Case 3 was a private NEI which had nursing schools in different provinces (Gauteng, Western Cape and KwaZulu-Natal), and from which the researcher believed the participants would offer a more diverse experience.

Case 4 was grouped as cases of interest, because these students had completed their training and had graduated from selected KZN NEIs. These students had contacted the researcher after completing the programme for participation in the study to share their experiences. Two SNWDs were in the R2175 programme and one was in the R683 programme. No student could be identified who had completed the R425 diploma programme.

### Data collection method

Ten individual semi-structured interviews were conducted with each SNWD, using an interview guide to ensure all aspects of the phenomenon were covered. They were conducted in a quiet place, depending on the location of the student and were digitally audiotaped with the participants’ prior written consent (Burns & Grove 2009:405). Analytical notes were also made during the interviews.

#### Data analysis

Data collection and analysis occurred simultaneously, using the case study protocol and study objectives to guide the data analysis process. Conventional content analysis was employed to analyse the data collected from interviews (Hsieh & Shannon 2005), following verbatim transcription. Conventional content analysis is utilised when there is a limited body of research or theory on the phenomenon being studied, as was the case of SNWDs in nurse training. Conventional content analysis enables the researcher to obtain direct knowledge from participants without any preconceived ideas. The resulting knowledge emanates from the views of participants and is grounded in the data. The data were divided into meaningful units for coding and categorising before theming. A report was written on the themes and forwarded to the supervisor for comments.

### Context of the study

This study was conducted in one public university and in private NEIs in KwaZulu-Natal, SA, during December 2016 to August 2017.

### Potential benefits and hazards

Gatekeeper permission was obtained from each individual NEI and the health facility in which the graduate nurses were employed. Individual consent was obtained from each participant for participation in the study and for having their voice recorded. Participants were informed that there was no monetary gain for participating in the study but that future SNWDs would benefit from the development of policy guidelines in nurse training. They were reassured that their names would be kept confidential, and their right to self-determination, privacy, anonymity, confidentiality, fair treatment and protection from harm and discomfort would be respected (Emanuel et al. 2004).

### Recruitment procedure

After the preliminary analyses of the survey questionnaires from phase 1, the researcher contacted the private NEIs that had SNWDs to obtain their contact details. The SNWDs were contacted telephonically to explain the nature of the study and potential participation. Interviews were then scheduled at a date, time and venue convenient to each consenting participant. They were informed that participation was voluntary and that they could withdraw at any time without penalty.

### Trustworthiness

Trustworthiness refers to the quality of the completed research study and its findings (Lincoln & Guba 1985:290). Lincoln and Guba (1985:301) use the terms ‘credibility’, ‘dependability’, ‘confirmability’ and ‘transferability’ to describe trustworthiness. Trustworthiness of the data was ensured by member checking, peer debriefing and prolonged engagement.

### Credibility

Credibility addresses confidence in the truth of the research findings and the data analysis to confirm the focal point of the research. The researcher used the following activities to ensure the study credibility (Lincoln & Guba 1985).

#### Member checking

Member checking was done after data collection and analysis, where the researcher provided participants with feedback to ensure the transcribed data were true representations of the lived experiences of each SNWD.

#### Peer debriefing

Peer debriefing occurs when members not involved in the study meet to discuss the methods and findings of the study. In this study, the researcher met with the supervisor for this purpose.

#### Prolonged engagement

In each interview, the researcher engaged with the participant in the field for a prolonged period. The researcher ensured immersion in the data during transcribing and analysing the data over a period of 9 months, listening to the tapes repeatedly to obtain in-depth understanding.

### Dependability

Dependability refers to the ability of the researcher to capture and analyse the data over time in its true form (Graneheim & Lundman 2004). In this study, dependability was ensured by conducting the research under supervision and guidance of an experienced researcher. Measures to ensure credibility were also employed to ensure dependability (Lincoln & Guba 1985:316).

### Transferability

Possible transferability to similar contexts was enhanced by providing a clear indication of the culture and context of the way participants were selected and of the processes used for data collection and analysis (Graneheim & Lundman 2004). In keeping with case study protocol, a database was created during data collection for easy retrieval of steps followed in the study and information.

### Confirmability

Confirmability was ensured by the researcher providing findings that are accurate and can be traced back to the original data sources. The researcher used direct quotes as expressed by the participants in the study (Holloway & Wheeler 2010:303).

### Thick description

The researcher provided a thick description to give the reader a ‘sense of being there’ and chose meaningful units to ensure that the findings portrayed the voice of the participants and not the researcher’s own bias or perceptions (Lincoln & Guba 1985). Following data collection and analysis, a full report on the findings was forwarded to the supervisor for feedback and comment.

### Ethical considerations

Ethical approval was obtained from University of KwaZulu-Natal Ethics committee (reference number: HSS/1367/015D). Individual consent was obtained from each participant prior to the interview to participate in the research and to have the interview tape-recorded. Respondents were reassured that their names would be kept confidential and their right to self-determination, privacy, anonymity, confidentiality, fair treatment and protection from harm and discomfort were respected (Burns & Grove 2009:715; Emanuel et al. 2004:89). There was no financial gain for participating in this research, but respondents were reimbursed for travelling to a neutral venue for the interview and a light lunch.

## Results and discussion

This article focuses specifically on eliciting the views of students and qualified nurses with disabilities who graduated at NEIs in KwaZulu-Natal and whose primary demographic data is indicated in Table 1.

**TABLE 1:** Demographic profile of the participants.

No.	Participant pseudonyms	Age	Gender	Year of study	Nursing programme	Type of disability
**Case 1**						
1	Mathew	40	Male	2nd	Bachelor of Nursing Science Degree	Vision impaired
2	Katy	24	Female	Completed in 2014	Bachelor of Nursing Science Degree	Hearing impaired
**Case 2**						
3	Rachael	23	Female	2nd	Enrolled nurse programme	Limping gait
4	Martha	24	Female	2nd	Enrolled nurse programme	Missing digit
5	Mary	44	Female	2nd	Bridging programme	Disabled hand
**Case 3**						
6	Mark	34	Male	2nd	Bridging programme	Dyslexia
7	John	27	Male	Completed in 2014	Bridging programme	Hand tremors
**Interesting cases**						
8	Isobel	41	Female	2nd	Bridging programme	Hearing impaired
9	Pam	44	Female	Completed in 2016	Enrolled nurse programme	No thumbs
10	Esther	53	Female	Completed in 2016	Enrolled nurse programme	Arthritis

### Biographical information

Gender representation of the overall participants was 70% (*n* = 7) women and 30% (*n* = 3) men, confirming that nursing is a female-dominated profession. According to Philips and Miltner (2015), nurses are an ageing population, and in this study majority of the participant were over the age of 30 years, making it likely that the prevalence of disability in nursing could increase.

### Experiences of student nurses who have disabilities

Three themes and eight related sub-themes emerged from the research findings on the experiences of students and nurses with disabilities who graduated from NEIs (see Table 2). As this is part of a much larger study, only selected findings of theme 1 (Disclosure), theme 2 and theme 3 are reported here.

**TABLE 2:** Themes and sub-themes: Experiences of students and qualified nurses with disabilities in nurse training programmes

Themes	Sub-themes
1. Diverse experiences of being a SNWD in nurse training	1.1: Personal experiences of fear and isolation
	1.2: Experiences of SNWDs as being ‘dis-ABLED’
	1.3: SNWDs’ experiences of disclosure
2. Experiences of SNWDs in the academic environment	2.1: Nurse educators as playing a pivotal role in SNWDs’ learning
	2.2: SNWDs’ experiences of lack of support and reasonable accommodation as organisational unpreparedness
	2.3: SNWDs’ experiences of barriers to effective communication
3. Student experiences during clinical placement	3.1: SNWDs’ experiences of providing cultural congruent care
	3.2: SNWDs’ experiences of collaboration with other healthcare workers

SNWD, student nurses who have disabilities.

#### Sub-theme 1.3: Experiences of disclosure of student nurses who have disabilities

Disclosure in this study refers to SNWDs voluntarily informing NEIs about their disability, either on admission or during the nurse training programme. In the present study, none of the nine SNWDs disclosed having a disability on admission to the NEI. The reason presented for this was fear that they may not be accepted onto the nurse training programme. Two students went through their entire training without disclosing their disability and two students reported that a lecturer noticed the disability. This study finding corresponds with the findings by Goode (2006) in which students chose not to disclose invisible disabilities because of stigma attached to disclosure (Elcock 2014).

Students with visible disabilities developed a tendency to ‘hide’ the affected limb, which was an unintentional force of habit. Some students had hidden disabilities such as missing digits and clubbed fingers which were easy to hide. This resulted in educators and healthcare professionals (HCPs) not being aware of the students’ disabilities or finding out very late during the training (Case 2, Martha, female). Morris and Turnbull (2006) highlight the importance of disclosure to invoke entitlement to reasonable accommodation because failure to disclose or delayed disclosure is a potential barrier to learning. Mosia and Phasha (2017) add that failing to disclose disability timeously on recruitment resulted in delay in providing reasonable accommodation and support to SNWDs.

Different reasons were presented by those students who decided to disclose. For example, two hearing-impaired students disclosed their disability after being accepted onto the programme because they feared that, ‘if a patient called them from behind they may not hear the patient which may result in potential medico-legal hazards’ (Case 1, Katy, female). However, educators revealed that no medico-legal hazards were reported during clinical placement of SNWDs to nurse patients. A recent study by Neal-Boylan and Smith (2016) supports these findings that there are no reported cases of medico-legal hazards occurring as a result of patients being nursed by SNWDs.

#### Theme 2: Experiences in the academic environment of student nurses who have disabilities

For some SNWDs, principal factors influencing their experience in the nurse training programme were the role of the nurse educator, communication, provision of support and reasonable accommodation.

#### Sub-theme 2.1: Nurse educators as playing a pivotal role in learning of student nurses who have disabilities

The critical role of the nurse educator was a recurring theme throughout the interviews with SNWDs and was perceived as important for the success of students in nurse training. For example, SNWDs experienced positive reactions from lecturers who were aware of their disability, usually through disclosure by the SNWD. For example, some lecturers provided notes to facilitate the lectures and a seat reserved in the front row for hearing-impaired students. Previous study findings substantiate that the lack of teaching aids, notes on the board or use of PowerPoint presentations makes it difficult for visually and hearing-impaired students to understand lectures (Emong & Eron 2016). The pivotal role of nurse educators in enhancing the learning and progress of SNWDs is supported by Mutanga (2017).

Other SNWDs reported negative experiences as follows: ‘some educators just wanted to teach, so they did not want to bother them with their problems’ (Case 1, Katy, female). They, therefore, intentionally did not disclose their disability to avoid discrimination. Neal-Boylan and Smith (2016) revealed that an academic environment where SNWDs feel supported encourages early disclosure.

SNWDs further reported that their experiences sometimes depended on a lecturer’s mood, varying on different days. Lecturers were very accommodating and supportive on some days, but not on others. For example, when they appeared to be in a ‘bad mood’, they did not allow SNWDs to ask questions in class, nor did they want to repeat themselves, as related in the following quote:

‘Or maybe [*the lecturer*] was just shouting at me. Or maybe when you ask something, they just look at you, they don’t respond to you. So, it means on that day, she’s not in the right mood.’ (Interesting case, Isobel, female)

SNWDs experienced these non-verbal cues and facial expressions of lecturers as expressing lack of acceptance regarding their disabilities in a class of non-disabled students. They also experienced frustration when lecturers forgot they had a disability, as related in the following excerpts:

‘Maybe the lecturer asks you the question, and then I answered, “I did not hear the question” and then she [*the lecturer*] said, “how come, why are you not serious to concentrate or to listen when I’m talking?”. (Interesting case, Isobel, female)

‘Sometimes the lecturer will walk around the class while teaching and I will not hear what she said, because I need her to be in front of me, because I also lip read.’ (Case 1, Katy, female)

Similar findings were cited by Bell et al. (2016) that hearing-impaired students needed to lip read. Overall, lecturers were instrumental in the students’ progress towards meeting their course objectives. However, some less helpful experiences ran parallel to this support by lecturers, posing challenges to teaching and learning, and hindering the students’ progress.

#### Sub-theme 2.2: Lack of support and reasonable accommodation because of organisational unpreparedness

Granting that South African Higher Education (SAHE) has relevant policies and frameworks aimed at promoting the inclusion and participation of SWDs in higher education, these policies are not aimed at professional-based courses requiring a practical component such as nurse training. Hence, in this study, support and reasonable accommodation depended on the educator, which varied at each NEI. This may be because of the innate nature of nurses being caring, despite the absence of clear policy guidelines for nurse training. For example, the following comment indicates lack of accommodation measures for students with mobility impairments who had difficulty accessing lectures on the first floor:

‘The first week I used crutches, it was difficult for me but at the same time, the tutors were waiting for me at the top, I need to go up and I need to have my bag. Because she’ll [*lecturer*] be waiting for me at the top. They don’t even have a ramp.’ (Case 2, Rachael, female)

Similar findings were revealed by Hall and Belch (2000), that SWDs experienced difficulty accessing lecture venues. But it is significant to note that structural challenges are beyond the control of educators.

One NEI enlisted the assistance of a scribe for a student with a disability, but extra time during the examinations was initially denied. The student had explained that he needed to repeat himself, sometimes more than once. The student reported that this was time-consuming and expressed his frustration as follows:

‘Then the issue when somebody is writing for you, you got the answers, but the scribe is slow, you must wait for that person to finish the sentence. Now you’ve lost what you wanted to say. And then you have to think back and repeat yourself.’ (Case 3, John, male)

The current study confirms that support and reasonable accommodation was provided to a limited extent in selected NEIs; for example, while some provided additional time for tests and examinations, others did not. Similar findings by Tee et al. (2010) highlighted 20% more contact time needed by SWDs than their non-disabled peers to learn, rehearse and achieve proficiency in clinical skills. Provision of support in some NEIs was situational rather than systemic, depending largely on the individual lecturer. Similarly, Gelbar et al. (2015) found that support for SWDs was left to the discretion of lecturers and that students’ experience of this was ‘feeling left behind’. One explanation for the lack of support could be educators’ lack of understanding or training in disability and support measures (Mosia & Phasha 2017).

#### Sub-theme 2.3: Barriers to effective communication

Impaired communication between key stakeholders was a barrier to successful inclusion and participation of SNWDs in nurse training, evidenced in the soft tone of lecturers which made it difficult for hearing-impaired students to hear. Soft speech in communication between doctors and nurses in theatre, where masks are worn, was a frequent problem for hearing-impaired students, as they were unable to lip read. Lack of communication between lecturers and personnel at clinical facilities made it difficult for SNWDs to adjust. They reported that with each monthly changeover, they would be rotated to a new ward where they had to re-explain themselves to different members of staff from day to day and sometimes repeatedly to the same members of staff. In some countries, such as the United Kingdom, there is evidence that support provided in higher education is extended to fieldwork, where students graduating into professions need to practise to gain experience (Tee et al. 2010). A survey of the South African literature by Botham and Nicholson (2014) showed lack of evidence suggesting similar practices in SAHE.

#### Theme 3: Students’ experience in the clinical facilities

SNWDs’ experiences highlighted their concerns in collaborating with other HCPs and the advantage of their being able to provide culturally congruent care because of their unique nature in also having a disability.

#### Sub-theme 3.1: Experiences of providing culturally congruent care by student nurses who have disabilities

SNWDs often did not perceive themselves as ‘disabled’. Rather, they perceived themselves as able-bodied, but with some limitations. SNWDs expressed this as having to teach their ‘able-bodied’ peers that they considered themselves to be just as competent and safe as able-bodied students. They sometimes perceived themselves as performing better in the clinical environment, as expressed in the following comment:

‘I was working in theatre, and I was the one who was teaching them how to suture. They didn’t know, none of them they knew how to suture.’ (Case 2, Mary, female)

SNWDs also perceived themselves as being able to relate to patients better, as they had first-hand experience with physical impediment, thus providing congruent nursing care. As cited by Marks (2007), SNWDs can potentially improve nursing care and provide culturally congruent care because of their appreciative understanding of disability. In addition, SNWDs reported that they could identify with people with disabilities (Marks 2007).

Although SNWDs’ experiences of nursing patients during clinical placement indicated that patient care was not affected by the students having disabilities, they did report that patients sometimes mocked them, as evidenced in comments such as, ‘Oh, you also a patient, why you nursing me?’ (Case 2, Rachael, female). This could be the result of innate expectations by patients that nurses who are supposed to be taking care of patients need to be well themselves.

#### Sub-theme 3.2: Experiences of collaboration with healthcare professionals by student nurses who have disabilities

SNWDs experienced a range of support and reactions from HCPs during clinical placement. While some HCPs were very accommodating and supportive, others sometimes forgot that students had a hearing impairment and were annoyed when SNWDs asked them to repeat themselves, as reported in the following excerpt: ‘The sister would ask “why, just why you asking every time?”’ (Interesting case, Isobel, female).

Some SNWDs reported challenges in performing clinical procedures because of lack of reasonable accommodation, such as regular rest breaks after several hours of standing. They noted, however, that practices improved once the ward sister had a further opportunity to supervise the student, at which point she ‘recognised the student’s worth and disability and knew that she wasn’t just looking for excuses to sit’ (Case 2, Rachael, female).

Generally, SNWDs found the clinical facilities unprepared because of lack of policies to guide their practice and accommodation, and, consequently, experienced various difficulties such as having to constantly remind HCPs of their disabilities.

## Limitations of the study

This study was conducted in private NEIs only and therefore ought to be interpreted with caution, rather than generalised to all NEIs in SA. The author is a novice researcher, who conducted this study as a requirement for her doctoral degree.

## Recommendations

It would be beneficial for each NEI to develop internal policy guidelines for integrating SNWDs. NEIs have tended to rely on broader university and higher education policy, procedures and support that may not be beneficial to SNWDs or to the NEIs when applied to nurse training because nursing is practice-based. Student nurses are required to complete clinical practice hours in clinical areas created for able-bodied learners, whereas wider university policies are mostly designed to accommodate classroom-based learning. The researcher therefore recommends developing policy guidelines tailored to nurse training programmes.

Further recommendations are the following:

- Appointment of a Disability Liaison positioned to engage all those involved in nursing education to support SNWDs and ensure that accommodation and adjustments made in clinical facilities afford an opportunity for them to apply theory to practice.
- Development and implementation of a disability unit specifically for nursing, designed to assist and support SNWDs and academics in integrating SNWDs in NEIs training programmes. While it may not be feasible for each private NEI to have its own disability unit because of the small number of students they enrol compared to a university, a possible alternative would be to enlist the help of a central disability unit to assist both students and academics.

## Conclusion

The study findings confirm increasing numbers of SNWDs in nurse training programmes in selected private NEIs in KwaZulu-Natal, SA. It is significant to note that none of the SNWDs disclosed their disability on admission for fear of not being accepted onto the nurse training programme. For this reason, the researcher concludes that the actual number of SNWDs remains unknown. Exploring experiences of SNWDs highlighted their first-hand experiences and the daily challenges they are faced with. What this study did not explore, however, was whether the challenges were related to the non-disclosure, or late disclosure, of these disabilities. A follow-up study exploring experiences of SNWDs who have been afforded an enabling environment will therefore be of importance to confirm the benefits of disclosing early in the programme and thus be afforded maximum support. Such literature will be imperative to provide information that can be used to inform future research and practice to ensure SNWDs are not discriminated against. One way of doing this is to provide support and reasonable accommodation to SNWDs.

Provision of reasonable accommodation in this study occurred on a trial-and-error basis, depending on best practices and on individual lecturers. Initiatives, such as those provided by the university examination units, of providing examination scripts with large, bold print were applauded for making it easier for students with vision impairment. This is possibly attributable to the existence and input of a disability unit, these being very functional in these institutions. These initiatives were, however, generic and not specific to the SNWDs. Challenges were still experienced in the clinical environment, where these learners had to read doctors’ scripts and nursing notes. This is an indication that NEIs need to work with other relevant stakeholders, such as the Department of Health, to provide support for these students during their training, and beyond, when they complete their training and become professionals. Even though nurse educators were willing to support and integrate SNWDs in nurse training, lack of policy guidelines made the process difficult. The study concludes that nurse educators play a pivotal role in assisting and supporting SNWDs towards successful completion of courses. Negative experiences included lack of communication between nurse educators and HCPs in the clinical facilities regarding student disability and accommodation. Students experienced it as frustrating to constantly have to remind them of their disability. There are merits in training SNWDs, as they can be role models, providing culturally competent care, as they have first-hand experience of having a disability.

Significant shortcomings remain in the experiences of SNWDs in KwaZulu-Natal NEIs. Differences exist between NEIs in the methods they employ to address disability. This results in a very fragmented accommodation of SNWDs, leaving them isolated from transformation that should currently be taking place in NEIs in the province. It is not enough to assume that the NEIs can function effectively within broader university or higher education policy without specific attention to SNWDs needs in both the clinical and academic settings. The development of policy guidelines will thus benefit SNWDs and any nurse who becomes disabled.
